# Targeted Metabolomics of Serum Acylcarnitines Evaluates Hepatoprotective Effect of Wuzhi Tablet (*Schisandra sphenanthera* Extract) against Acute Acetaminophen Toxicity

**DOI:** 10.1155/2013/985257

**Published:** 2013-02-04

**Authors:** Huichang Bi, Fei Li, Kristopher W. Krausz, Aijuan Qu, Caroline H. Johnson, Frank J. Gonzalez

**Affiliations:** ^1^School of Pharmaceutical Sciences, Sun Yat-sen University, 132 Waihuandong Road, University City, Guangzhou 510006, China; ^2^Laboratory of Metabolism, Center for Cancer Research, National Cancer Institute, NIH, Bethesda, MD 20892, USA; ^3^Center for Metabolomics and Mass Spectrometry, The Scripps Research Institute, La Jolla, CA 92037, USA

## Abstract

Possible prevention and therapeutic intervention strategies to counteract acetaminophen (APAP) hepatotoxicity would be of great value. Wuzhi tablet (WZ, extract of *Schisandrae sphenanthera*) possesses hepatoprotective effects against hepatitis and the hepatic dysfunction induced by various chemical hepatotoxins. In this study, the protective effect of WZ on APAP-induced hepatic injury was evaluated and targeted metabolomics by LC-MS-based metabolomics was used to examine whether WZ influences hepatic metabolism. The results demonstrated significant hepatoprotection of WZ against APAP-induced liver injury; pretreatment with WZ prior to APAP administration blocks the increase in serum palmitoylcarnitine and oleoylcarnitine and thus restores the APAP-impaired fatty acid **β**-oxidation to normal levels. These studies further revealed a significant and prolonged upregulation of the PPAR**α** target genes *Cpt1* and *Acot1* by WZ mainly contributing to the maintenance of normal fatty acid metabolism and thus potentially contributing to the hepatic protection of WZ against APAP-induced hepatic toxicity. Taken together, the current study provides new insights into understanding the hepatoprotective effect of WZ against APAP-induced liver toxicity.

## 1. Introduction

Acetaminophen (APAP), also known as paracetamol and N-acetyl-p-aminophenol, is the most frequently used and readily available over-the-counter (OTC) analgesic and antipyretic agent in the world. It is found in many OTC combination drugs (contained in >100 products) and its use has achieved great popularity, with more than 1 billion tablets sold annually in the United States [1]. While APAP is relatively safe at therapeutic doses, it can cause hepatotoxicity and acute liver failure in high doses, in the case of deliberate or accidental overdose. Ingestion of high doses of APAP is the most common cause of acute liver failure in the United States and worldwide [2]. Therefore, research into possible prevention and therapeutic intervention strategies to counteract APAP toxicity are essential and of clinical importance. Over the past four decades, numerous efforts have been undertaken to elucidate the molecular mechanism of APAP toxicity. Some of these mechanisms are well established showing that the toxicity is initiated by accumulation of the toxic reactive metabolite *N*-acetyl-p-benzoquinone imine (NAPQI). This metabolite can trigger subsequent hepatic toxicity by covalently binding with cellular proteins and/or by elevating reactive oxygen species (ROS) leading to apoptosis and cellular necrosis [3]. Recent developments in metabolomics have provided advanced analytical platforms to obtain more insights into the APAP-induced hepatotoxicity. A nuclear magnetic resonance- (NMR-) based metabolomics study demonstrated that APAP can cause disruption of carbohydrate and lipid metabolism [4, 5], while a capillary-electrophoresis-mass-spectrometry- (CE-MS-) based study identified ophthalmic acid as a novel biomarker of APAP toxicity [6]. Additionally, a recent study using liquid-chromatography- (LC-) MS-based metabolomics revealed novel drug and endogenous metabolites associated with APAP-induced hepatotoxicity [7]. Furthermore, LC-MS-based serum metabolomics of APAP-induced hepatotoxicity in wild-type and *Ppar*-null mice revealed that CYP2E1-mediated metabolic activation and oxidative stress following APAP treatment can cause irreversible inhibition of fatty acid *β*-oxidation, and indicated that acylcarnitines function as early biomarkers for monitoring the APAP-induced hepatotoxicity [8]. Inhibition of fatty acid *β*-oxidation through suppression of peroxisome proliferator-activated receptor alpha (PPAR*α*) was established as a major mechanism of APAP-induced hepatotoxicity. An increase of acylcarnitines was observed, and the PPAR*α* target gene uncoupling protein 2 (UCP2) protected against elevated ROS generated during APAP toxicity. This suggested that induction of UCP2 may also be a general mechanism for protection of mitochondria during fatty acid *β*-oxidation [8, 9].

 For the clinical importance of APAP, research into possible prevention and therapeutic intervention strategies to counteract APAP toxicity are therefore essential. Various herbs and herbal products are believed to have liver protective functions and are widely used in clinical practice worldwide [10–13]. *Schisandrae sphenanthera *(Nanwuweizi) is the dried ripe fruit of *Schisandra sphenanthera* Rehd. et Wils. It is historically listed among China's most important herbs to protect and improve the function of the liver, kidney, and heart, and it is now indexed in the Pharmacopoeia of China. Wuzhi tablet (WZ, registration number in China: Z20025766) is a preparation of an ethanol extract of *Schisandra sphenanthera*, which contains 7.5 mg Schisantherin A per tablet. Its major active chemical constituents include Schisandrin A, Schisandrin B, Schisandrin C, Schisandrol A, Schisandrol B, Schisantherin A, Schisantherin B, and others [14]. The whole extract and components in the extract have been shown to possess hepatoprotective effects against viral or chemical hepatitis and the hepatic dysfunction induced by various chemical hepatotoxins such as carbon tetrachloride [15–18]. However, there have been no studies to evaluate the effect of WZ on APAP-induced liver injury. Biological studies indicate that some schisandrins in the extract can enhance the mitochondrial hepatic glutathione (GSH) antioxidant/detoxification system and induce heat shock proteins in mice [17–19]. Yet many aspects of the mechanisms underlying the hepatoprotective action of WZ are not clearly defined or remain unknown.

 In the current study, the protective effect of WZ on APAP-induced hepatic injury was evaluated and LC-MS-based targeted metabolomics of serum acylcarnitines was carried out to examine the influence of WZ on mitochondrial function and fatty acid *β*-oxidation. Furthermore, PPAR*α* target genes indicated alteration in fatty acid metabolism were further characterized to uncover the relationship between fatty acid *β*-oxidation, PPAR*α* activation, and the hepatoprotective effect of WZ against APAP-induced toxicity.

## 2. Materials and Methods

### 2.1. Chemicals

APAP, palmitoylcarnitine and chlorpropamide were purchased from Sigma-Aldrich (St. Louis, MO). Myristoylcarnitine, oleoylcarnitine, and palmitoleoylcarnitine were obtained from the Metabolic Laboratory, Vrije Universiteit Medical Center (Amsterdam, The Netherlands). Wuzhi tablet (batch no.: 110711) was manufactured and supplied by Fang Lue Pharmaceutical Company (Guangxi, China) under GMP guidelines. This 70% ethanol extract of *Schisandra sphenanthera* was formulated with starch, carboxymethyl starch sodium, and magnesium stearate into tablets. The final product meets the China SFDA standard (YBZ14932006) and has been quantified to 7.5 mg Schisantherin A per tablet by HPLC analysis. In addition, content of major active constituents in the extract including Schisandrin A, Schisandrin B, Schisandrin C, Schisandrol A, Schisandrol B, and Schisantherin A were further identified and determined using a validated LC-MS/MS analysis. HPLC grade solvents were purchased from Fisher Scientific (Hampton, NH). All the other chemicals and solvents were of the highest grade commercially available.

### 2.2. Animals and Sample Collection

Male 6-week-old C57BL6 mice (Jackson Laboratories, Bar Harbor, ME) were maintained in a NCI animal facility under a standard 12 h light/12 h dark cycle with food and water* ad libitum*. All animal studies were approved by the NCI Animal Care and Use Committee. Mice were randomly divided into four groups: untreated control group, WZ-treated group, APAP-treated group, and WZ/APAP-treated group. For the WZ pre-treated mice, the tablet was crushed to a powder, dissolved in saline solution, and administered by gavage (i.g.) at doses of 700 mg/kg seven times with an interval of 12 h; the control group and APAP group were gavaged with saline solution. The dosage of WZ used in the current study was calculated based on a reported hepatoprotective dose of 500 mg/kg in rats [16] with correction for body surface difference between rats and mice and was estimated as the dose of 80 mg/kg used in clinical practice. For APAP treatment, APAP was dissolved in a saline solution and administered by intraperitoneal (i.p.) injection at doses of 400 mg/kg; and the untreated control group and WZ alone group were injected i.p. with saline solution. For the WZ/APAP group, 15 min after the seventh dose of WZ, APAP was given by i.p. injection. In a previous study [8], time-dependent changes in biomarkers of APAP-induced hepatotoxicity were observed and 2 h post-APAP was the key time point (e.g., acylcarnitines and GSH had most sensitive change at 2 h as observed in the study). Thus, another four groups were involved in the study to collect the serum samples 2 h after APAP treatment. Serum samples were collected and the liver was harvested, a portion of the liver was collected in 10% buffered formalin for histology and the remaining tissue was flash frozen in liquid nitrogen and stored at −80°C for further use.

### 2.3. Histological and Biochemical Assessment

Liver tissues were immediately formalin-fixed and paraffin embedded, sectioned, and stained with hematoxylin and eosin (HE) following a standard protocol. The HE-stained liver sections were examined using an Olympus BX41 microscope. APAP-induced liver injury was also evaluated by measuring serum aspartate aminotransferase (AST) and alanine aminotransferase (ALT) activities using VetSpec Kits (Catachem, Bridgeport, CT) following the manufacturer's instruction. GSH levels in liver after APAP and/or WZ treatment were measured using a glutathione assay kit (Sigma-Aldrich, St. Louis, MO).

### 2.4. UPLC-ESI-QTOFMS Analysis of Serum

Serum collected from mice were thawed and 20 *μ*L added to a microcentrifuge tube containing 180 *μ*L 67% aqueous acetonitrile and 5 *μ*M chlorpropamide (Internal standard). The samples were vortexed for 30 s each and centrifuged at 18,000 ×g for 15 min at 4°C to remove proteins and particulates. The supernatant was transferred to an UPLC vial (Waters Corp, Milford, MA). For all the above extractions, pooled samples were also made for quality control containing 5 *μ*L of each sample. Serum samples were randomized and analyzed. In brief, a 5 *μ*L aliquot of deproteinized serum sample was injected into a Waters ACQUITY UPLC system (Waters Corp, Milford, MA). A reverse-phase 50 × 2.1 mm ACQUITY 1.7 *μ*m BEH C18 column (Waters Corp, Milford, MA) was used to separate chemical components in serum. The mobile phase flow rate was 0.5 mL/min with an aqueous acetonitrile gradient containing 0.1% formic acid over a 10 min run. To avoid artifacts based on sample injection order, the order was randomized. Pooled, blank, and standard samples were injected after every five samples. MS was performed on a Waters QTOF-SYNAPT-MS operating in positive (ESI^+^) ionization mode. The capillary voltage and cone voltage were set to 3000 and 20 V, respectively. Source and desolvation temperatures were set at 120°C and 350°C, respectively. Nitrogen was used as both cone gas (50 L/h) and desolvation gas (600 L/h), and argon was used as collision gas. For MS scanning, data were acquired in centroid mode from 50 to 850 *m/z* and for MS/MS fragmentation of target ions the collision energy was ramped from 15 to 30 eV. 

### 2.5. Multivariate Data Analysis (MDA)

The mass spectral data were centroided, integrated, and deconvoluted to generate a multivariate data matrix using MarkerLynx (Waters Corp, Milford, MA). Peak picking, alignment, deisotoping, and integration were performed automatically by the software with optimized values of parameters such as mass tolerance, peak width, peak-to-peak baseline noise, intensity threshold, mass window, retention time window, and noise elimination level. The raw data were transformed into a multivariate matrix containing aligned peak areas with matched mass-to-charge ratios (*m/z*) and retention times. The data were normalized to peak area of the internal standard (chlorpropamide, which appeared at a retention time of 5.3 min, 277.041 [M+H]^+^). The multivariate data matrix was further exported into SIMCA-P+12 software (Umetrics, Kinnelon, NJ) and transformed by mean-centering and *Pareto* scaling to increase the importance of low abundance ions without significant amplification of noise. Either unsupervised principal components analysis (PCA) or supervised partial least squares-discriminant analysis (PLS-DA) models were constructed to analyze the serum data from APAP-treated/untreated and/or WZ-untreated/treated mice. Targeted acylcarnitines were identified by directly analyzing their ions showing considerable group discriminating power from the loadings scatter plot of PCA and PLS-DA models.

### 2.6. Quantitation of Palmitoylcarnitine and Oleoylcarnitine in Serum

Endogenous serum palmitoylcarnitine and oleoylcarnitine were quantitated using an Acquity UPLC H-class coupled to a XEVO G2 QTOFMS with Quantof technology (Waters Corp, Milford, MA). Standard calibration curves from 0 to 5 *μ*M were made for palmitoylcarnitine (*r* ^2^ = 0.9991) and oleoylcarnitine (*r* ^2^ = 0.9916) using authentic standards. Serums were diluted 1 : 20 in 67% aqueous acetonitrile containing 5 *μ*M of chlorpropamide (internal standard). The chromatographic conditions used were as listed above except that an additional 2 min equilibration step was needed with the H-class system. The XEVOG2 was operated in ESI+ with a capillary voltage of 3000 V and a sampling cone voltage of 30 V. The desolvation and cone gas flow were set to 850 and 50 L/H, respectively. The desolvation temperature was set to 450°C and the source temperature was 150°C. Data was acquired in centroid mode from 50 to 850 *m/z.* The samples were quantitated using TargetLynx (Waters Corp, Milford, MA) software by integrating peak areas of extracted ion chromatograms. The concentration of each metabolite was calculated from the normalized (with respect to internal standard) peak area using the standard calibration plot.

### 2.7. Assessment of Change in Fatty Acid Metabolism

The influence of APAP and/or WZ treatment in fatty acid metabolism was evaluated by measuring the levels of serum triglycerides and free fatty acids using colorimetric triglyceride assay reagent (Wako, Osaka, Japan) and free fatty acid assay reagent (Wako, Osaka, Japan). 

### 2.8. Gene Expression Analysis

Total RNA was extracted from frozen liver samples with Trizol reagent and quantified with NanoDrop (Thermo Scientific, Rockford, IL). cDNA was synthesized from 1 *μ*g total RNA using Superscript II reverse transcriptase kit (Invitrogen, Carlsbad, CA). For quantitative real-time PCR (qPCR) analysis, the primer pairs were designed using the Primer Express software (Applied Biosystems) and all primers were further validated using NCBI Primer-BLAST. The primer sequences have been published [8]. qPCR reactions contained 25 ng of cDNA, 150 nM of each primer, and 5 *μ*L of SYBR Green PCR Master Mix (Applied Biosystems, Foster City, CA) in a total volume of 10 *μ*L. All reactions were performed in triplicate on an Applied Biosystems Prism 7900HT Sequence Detection System (Applied Biosystems, Foster City, CA). Relative mRNA levels were calculated by the comparative threshold cycle method using *β*-actin as the internal control.

### 2.9. Statistical Analysis

All values were expressed as mean ± SD. Statistical analysis was performed by Student's *t*-test for unpaired data using GraphPad Prism 5 (GraphPad Software Inc., San Diego, CA). Difference was considered as significant if the probability (*P* value) was less than 0.05 (*P* < 0.05).

## 3. Results

### 3.1. WZ Pretreatment Protects against APAP-Induced Hepatotoxicity

**Figure 1:**
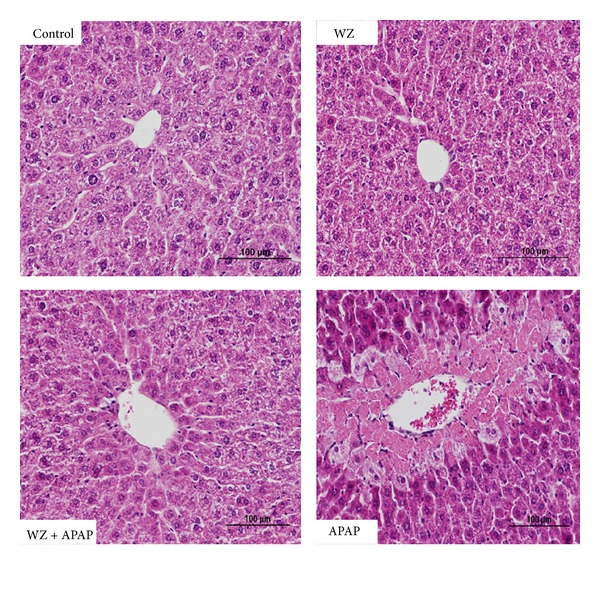
WZ protects against APAP-induced liver toxicity. WZ was administered to mice by gavage for 3 days prior to treatment with APAP. H&E staining of livers from control, WZ-treated, WZ/APAP-treated, and APAP-treated mice. Bars represent 100 *μ*m.

**Figure 2:**
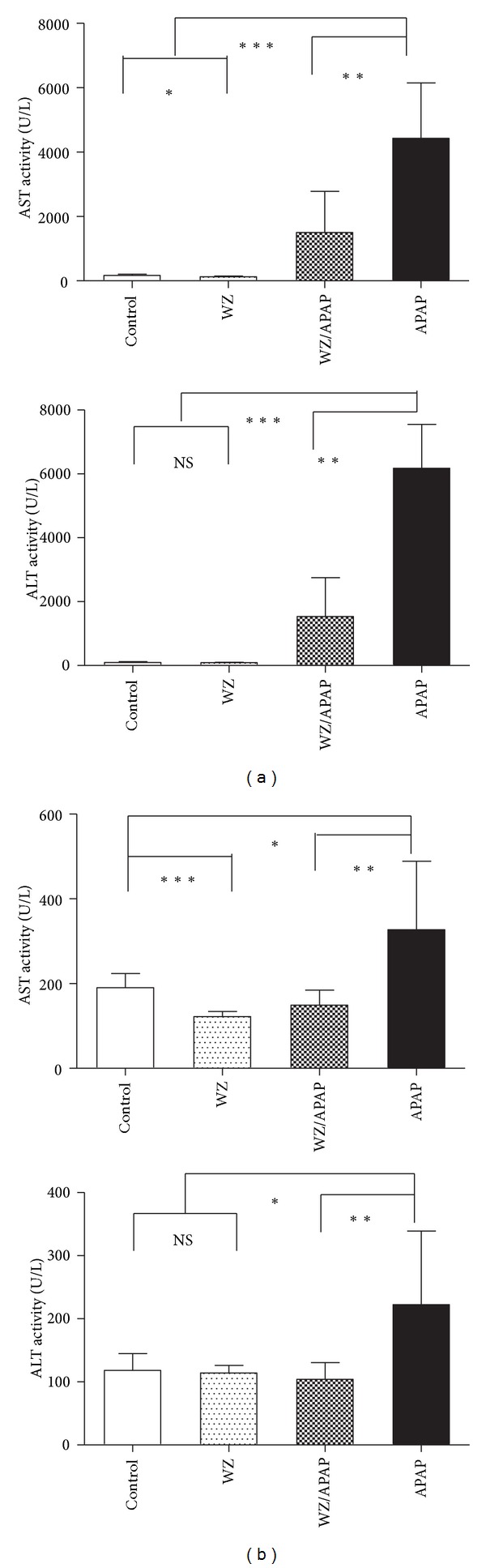
WZ protects against APAP-induced liver toxicity. Serum AST and ALT enzyme levels of 2 h (a) or 24 h (b) serum samples from control, WZ-treated, WZ/APAP-treated, and APAP-treated mice (mean ± SD, *n* = 5 in each group). *P < 0.05, **P < 0.005, ***P < 0.0001, NS: not significant.

**Figure 3:**
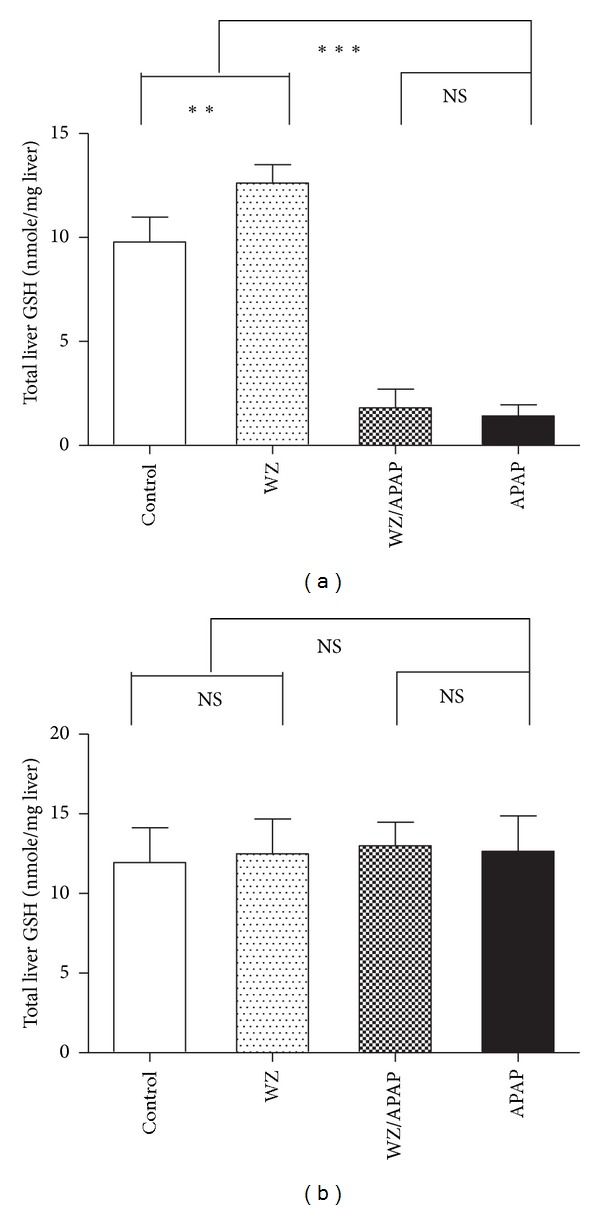
Total liver glutathione levels in control, WZ-treated, WZ/APAP-treated, and APAP-treated mice. Hepatic glutathione levels of 2 h (a) or 24 h (b) serum samples from control, WZ-treated, WZ/APAP-treated, and APAP-treated mice were measured by colorimetric methods (*n* = 5 in each group). *P < 0.05, **P < 0.005, ***P < 0.0001, NS: not significant.

To evaluate the protective effect of WZ on APAP-induced hepatic injury, liver histology and serum ALT/AST activity were assessed. Results from previous studies revealed that wild-type mice were susceptible to 400 mg/kg APAP [7, 8]. In this study, treatment with 400 mg/kg APAP for 24 hours resulted in massive hepatic toxicity as revealed by H&E staining of livers (Figure 1) and strikingly increased AST and ALT enzyme activities (Figure 2(a)). Pretreatment with WZ for three days before APAP treatment resulted in marked protection against APAP-induced hepatic injury. WZ-pretreated mice had no overt evidence of liver damage as revealed by normal liver histology (Figure 1) and significantly reduced AST and ALT activities (Figure 2(a)) compared with those of APAP-treated mice. At 2 h post-APAP treatment, WZ-pretreated mice were protected, as indicated by reduced AST and ALT levels (Figure 2(b)). On the other hand, WZ pretreatment alone reduced AST and ALT activities both at 2 h and 24 h post i.p.-APAP-dosing, as indicated by significant reductions in AST activity and a slight decrease in ALT levels compared to the control (Figures 2(a) and 2(b)). APAP toxicity can also be associated with depletion of GSH levels and thus the levels of total liver GSH were examined. Treatment with 400 mg/kg APAP for 2 h resulted in a decrease in total liver GSH levels (Figure 3(a)), that preceded the increase of AST and ALT activity. WZ increased GSH levels compared to the control, while WZ-pretreated mice still exhibited extensive GSH depletion at 2 h post-APAP treatment. This decrease was however partially restored with a slight increase in GSH levels. Consistent with previous studies [8], hepatic glutathione levels were recovered at 24 h of APAP treatment (Figure 3(b)), which was distinct from the changes in AST and ALT levels. 

### 3.2. Targeted Metabolomics of Serum Acylcarnitines Associated with WZ Protection against APAP-Induced Hepatic Toxicity

**Figure 4:**
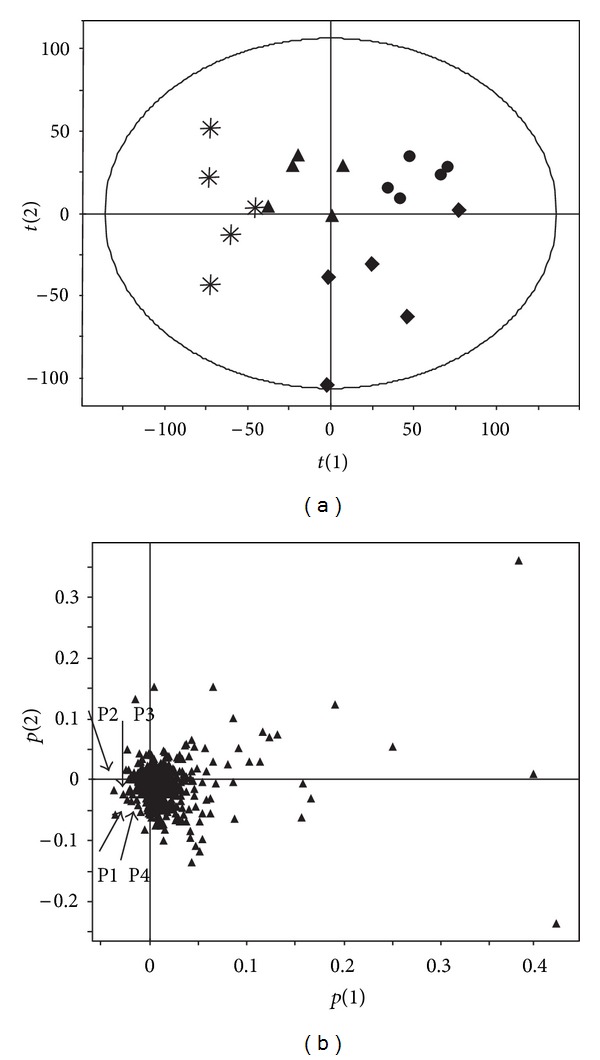
Identification of acylcarnitines as serum biomarkers of WZ protective effect on APAP-induced hepatic toxicity through metabolomic analysis. (a) Scores scatter plot of a PCA model on the serum metabolome of control (∗), WZ-treated (▲), WZ/APAP-treated (•), and APAP-treated (◆) mice. (b) Loadings scatter plot from the PCA analysis of serum from control, WZ-treated, WZ/APAP-treated, and APAP-treated mice. Data points representing palmitoylcarnitine (P1), oleoylcarnitine (P2), myristoylcarnitine (P3), and palmitoleoylcarnitine (P4) were labeled in the plot.

**Figure 5:**
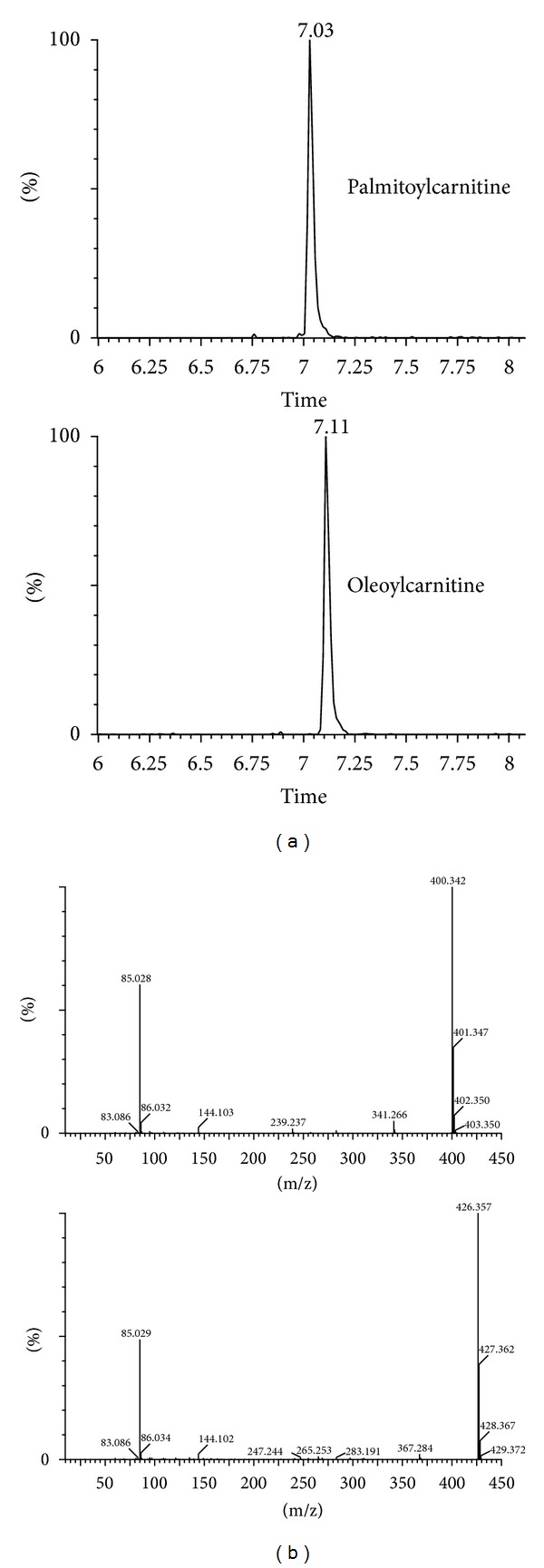
(a) Ion chromatograms of serum palmitoylcarnitine and oleoylcarnitine by extracting ions of 400.34^+^ and 426.35^+^. (b) LC-MS/MS spectra of ions of 400.34^+^ and 426.35^+^ in serum sample.

**Figure 6:**
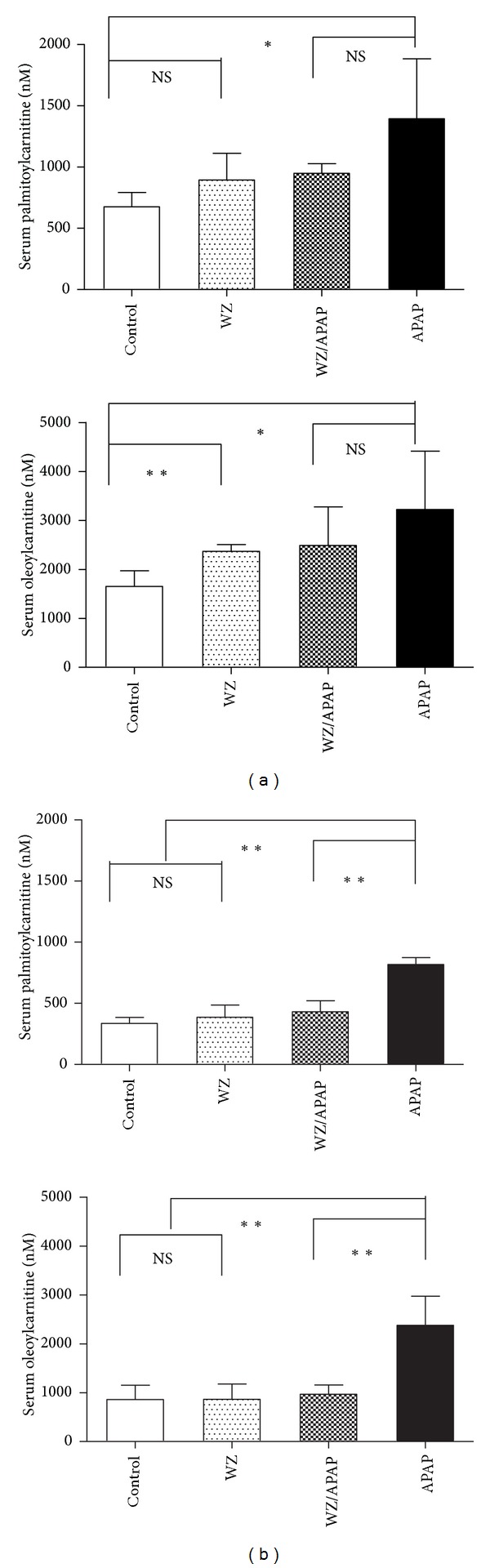
Quantitation of serum palmitoylcarnitine and oleoylcarnitine level in 2 h (a) and 24 h (b) serum samples from control, WZ-treated, WZ/APAP-treated, and APAP-treated mice (*n* = 5 in each group). *P < 0.05, **P < 0.005, ***P < 0.0001, NS: not significant.

**Figure 7:**
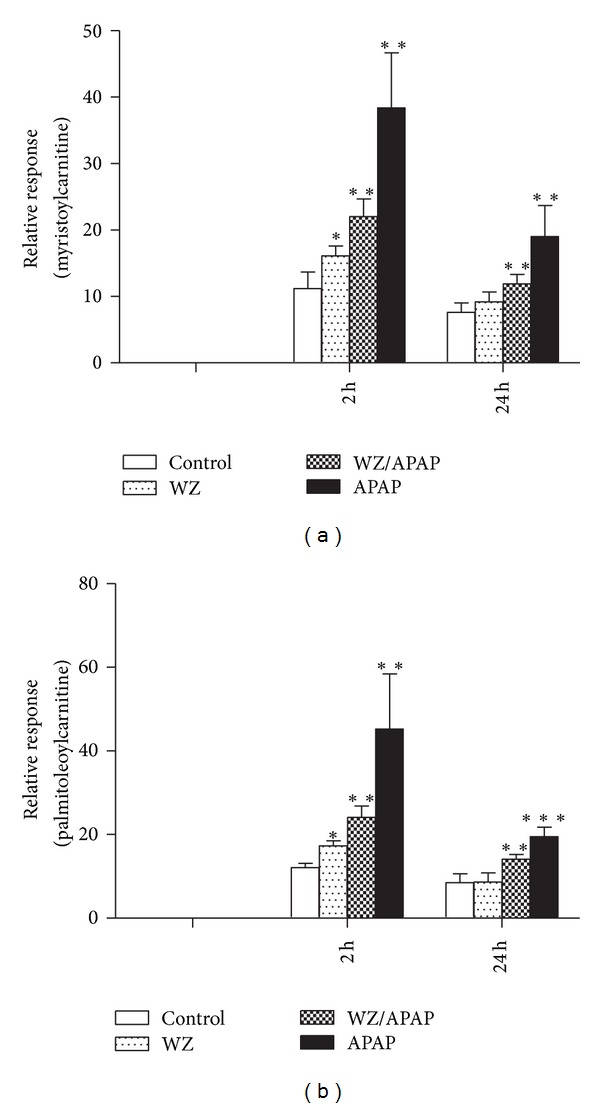
Relative response of serum myristoylcarnitine (a) and palmitoleoylcarnitine (b) levels in 2 h and 24 h serum samples from control, WZ-treated, WZ/APAP-treated, and APAP-treated mice (*n* = 5 in each group). *P < 0.05, **P < 0.005, ***P < 0.0001, NS: not significant.

Results from a previous study revealed that mitochondrial damage and inhibition of fatty acid *β*-oxidation is a major mechanism of APAP-induced hepatotoxicity; the toxicity is also associated with elevation of long-chain acylcarnitines in serum and acylcarnitines which can function as complementary biomarkers for monitoring the APAP-induced hepatotoxicity [8]. To further examine whether acylcarnitines could serve as indicative biomarkers for APAP toxicity and WZ-induced protective effects, targeted serum acylcarnitine metabolomics was carried out by a UPLC-ESI-QTOFMS system and characterized by MDA. Data extracted from the chromatograms and mass spectra of serum samples 24 h postdosing were processed by PCA and PLS-DA analysis. Sample distribution pattern in the scores scatter plot indicated that the mice in control group were clearly separated from all four mouse groups and the APAP-treated mice were also well separated from the other three groups (Figure 4(a)). This observation was consistent with the hepatotoxic status of this mouse groups. Further analysis of the loadings scatter plot of PCA and PLS-DA models showed that four ions with *m/z* of 426.35^+^, 400.34^+^, 398.32^+^, and 372.31^+^ were important contributors to the separation of APAP-treated mice from other three mice groups (Figure 4(b)). These four ions were also found to importantly contribute to the separation of different four groups by PCA and PLS-DA analysis of data matrix of serum samples 2 h postdosing. The fragmentation of these four ions resulted in very similar mass spectra, in which a carnitine fragment (*m/z* = 85.029^+^) and fatty acid moiety appeared. The identities of ions with *m/z* of 426.35^+^ and 400.34^+^ were confirmed after carrying out comparison of retention time and tandem mass spectra to authentic standards and were identified as oleoylcarnitine and palmitoylcarnitine, respectively (Figure 5). Ions with *m/z* of 398.32^+^ and 372.31^+^ were identified as palmitoleoylcarnitine and myristoylcarnitine by comparing the MS/MS fragments with that in a previous publication [7]. These are major long-chain acylcarnitine species in serum. Moreover, quantitation of oleoylcarnitine and palmitoylcarnitine showed that levels of palmitoylcarnitine and oleoylcarnitine 2 h after APAP treatment were higher than the control and the WZ/APAP-treated mice (Figure 6(a)). Twenty-four hours after 400 mg/kg APAP treatment, the oleoylcarnitine and palmitoylcarnitine levels were significantly higher than the corresponding control and the WZ/APAP-treated mice (Figure 6(b)). The relative response of palmitoleoylcarnitine and myristoylcarnitine had similar change as that of oleoylcarnitine and palmitoylcarnitine 2 h and 24 h after APAP treatment (Figure 7). Pretreatment with WZ prior to APAP administration blocked the increase in palmitoylcarnitine and oleoylcarnitine as indicated by direct quantification. Two-hour-post APAP and/or-WZ treatment, the level of acylcarnitines in each group was much higher than that of 24 h after APAP treatment suggesting that at 24 h after dosing the level of fatty acid *β*-oxidation was stronger than that of at 2 h. This might be due to a slight fasting challenge to the mice at 24 h postdosing in which fatty acid *β*-oxidation becomes the principal catabolic pathway for energy generation.

### 3.3. Changes in Fatty Acid Metabolism Associated with WZ Protection against APAP-Induced Hepatic Toxicity

**Figure 8:**
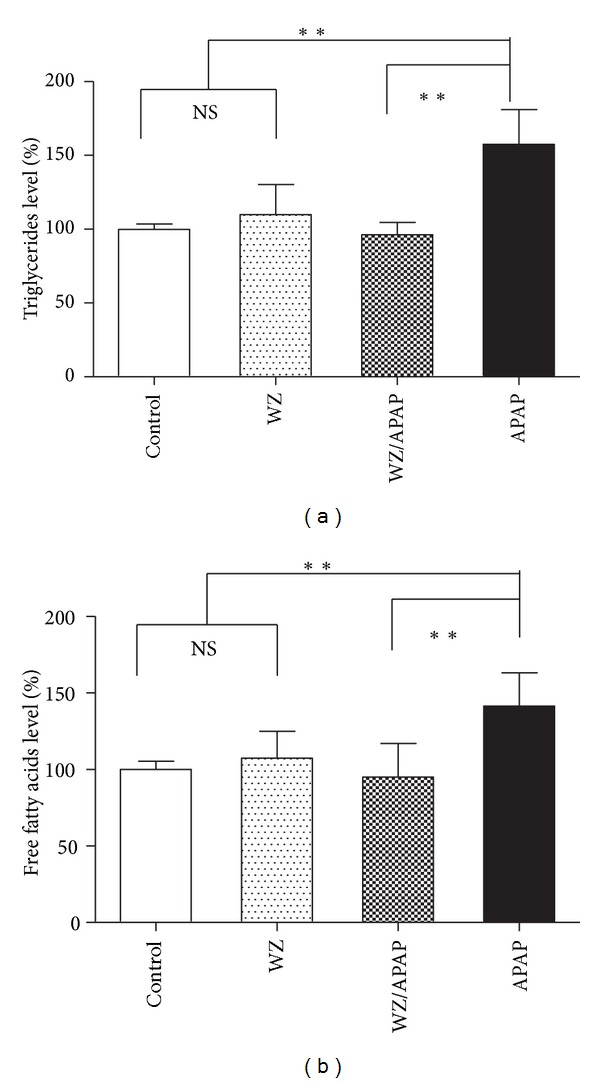
Influence of WZ and/or APAP on lipid metabolism. Triglyceride and free fatty acid levels in 24 h serum samples from control, WZ-treated, WZ/APAP-treated, and APAP-treated mice were measured by colorimetric methods. (a) Serum triglyceride level, (b) serum free fatty acid level (mean ± SD, *n* = 5 in each group). Triglyceride and free fatty acid levels in control mice were arbitrarily set as 100%. *P < 0.05, **P < 0.005, ***P < 0.0001, NS: not significant.

Inhibition of fatty acid *β*-oxidation was established as a major mechanism of the hepatotoxicity induced by APAP [8]. Since long-chain acylcarnitines are the essential intermediates of fatty acid *β*-oxidation, the changes of serum acylcarnitine levels have been associated with lipid metabolism disorders [20]. In a previous report [8], serum triglycerides (TG) and free fatty acid (FFA) levels were correlated with changes in serum acylcarnitine levels in 400 mg/kg APAP-treated mice. To correlate the acylcarnitine levels with the fatty acid metabolism in the WZ protection study, the levels of serum TG and FFA were examined. Consistent with the profile of acylcarnitines, the dramatic increases of serum TG and FFA only occurred in the APAP-treated mice while without significant changes in the other three groups (Figures 8(a) and 8(b)). This observation further confirmed that APAP at 400 mg/kg can severely disrupt the *β*-oxidation of fatty acids while WZ can recover this inhibition and maintain the normal lipid metabolism.

### 3.4. Different Responses of PPAR*α*-Targeted Genes to APAP/WZ Treatment

**Figure 9:**
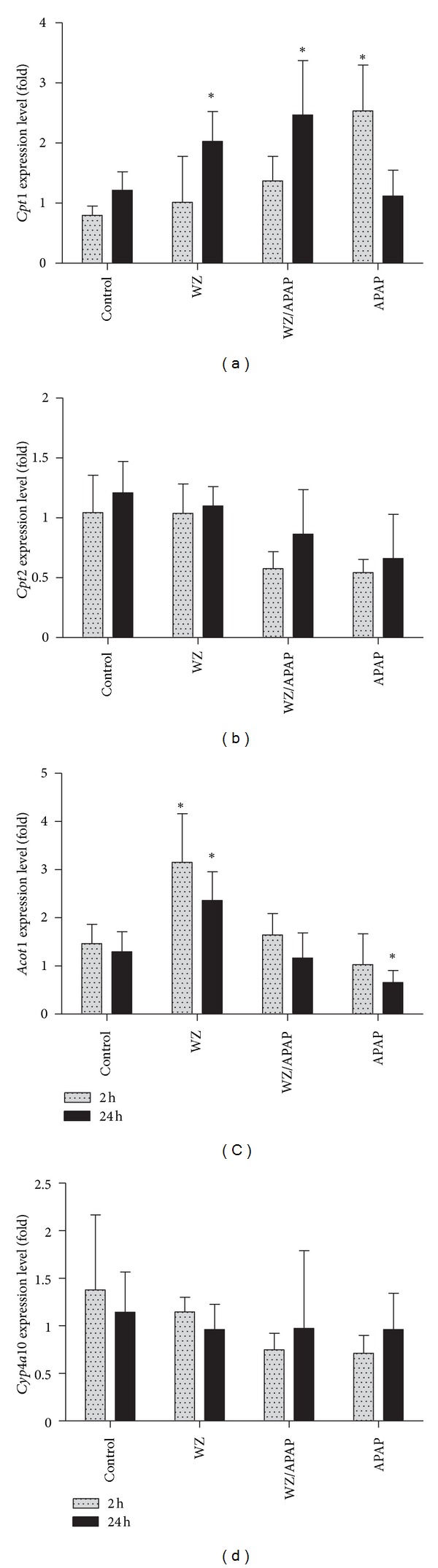
PPAR-targeted gene expression in livers from control, WZ-treated, WZ/APAP-treated, and APAP-treated mice. Liver samples were collected at 2 h and 24 h after APAP treatment. The gene expression levels were measured by real-time PCR and normalized by *β*-actin. Gene expression level in control mice was arbitrarily set as 1 (*n* = 5 in each group). *P < 0.05, compared to the control.

Previous studies revealed a direct connection between the accumulation of long-chain acylcarnitines and inhibition of the PPAR*α* function [8]. In this study, the correlation of PPAR*α* function and effect of WZ on APAP-induced acylcarnitine accumulation was examined by measuring the expression of four PPAR*α* target genes, including carnitine palmitoyltransferase I (*Cpt1*), carnitine palmitoyltransferase 2 (*Cpt2*), acyl-CoA thioesterase 1 (*Acot1*), and cytochrome P450 4A10 (*Cyp4a10*), in the livers. The increase of *Cpt1 *gene expression was observed in all APAP- and/or WZ-treated mice. However, the increased expression level of *Cpt1* was sustained for 24 h in WZ-treated groups while its expression in APAP-treated mice was reduced to basal level at the end of 24 h (Figure 9(a)). Compared to *Cpt1*, the response of *Cpt2* to WZ was less intense, not significantly different from control, and decreased to less than half of the basal level after APAP treatment (Figure 9(b)). *Acot1* was strongly induced by WZ and this induction was prolonged for 24 h while its expression in APAP-treated mice was not different from the basal level (Figure 9(c)). *Cyp4a10* expression was similar to the expression pattern of *Cpt2* with lower levels than that of the controls (Figure 9(d)). Overall, some differences among these gene expression patterns were found, and WZ exhibited inductive effects on both *Cpt1* and *Acot1*. These results indicate that the significant and prolonged upregulation of *Cpt1* and* Acot1 *by WZ mainly contributed to maintain the normal fatty acid metabolism and thus potentially contributed to the hepatic protection of WZ against APAP-induced hepatic toxicity. 

## 4. Discussion

The present study demonstrates a significant protective effect of Wuzhi tablet (*Schisandra Sphenanthera* extract) on APAP-induced liver injury, and targeted metabolomics of serum acylcarnitines showed a novel correlation with the hepatoprotective effect of WZ on APAP toxicity. Acylcarnitines are thus validated to serve as complementary indicative biomarkers for the WZ-induced hepatoprotective effect against APAP toxicity. Further analysis on PPAR*α* target genes indicated that the significant upregulation of *Cpt1* and *Acot1 *by WZ principally mainly contributed to maintain the normal fatty acid metabolism and thus potentially contributed to the hepatic protection of WZ against APAP-induced liver injury. 

 Various herbs and herbal products have been proven to process liver protective functions against APAP-induced hepatotoxicity [10–12, 21, 22]. However, there have been no studies to evaluate the effect of WZ on APAP-induced liver injury. In the present study, when WZ was administered prior to a toxic dose of APAP (400 mg/kg), mice were dramatically protected against hepatotoxicity, as revealed by H&E-stained liver sections showing no significant liver damage, and lower serum AST and ALT enzyme activity compared with mice only treated with APAP. WZ already exhibited a protective effect 2 h post-APAP treatment as evidenced by a significant decrease in AST and ALT levels. These results are in strong agreement with previous reports demonstrating the hepatoprotective effect of the whole *Schisandra Sphenanthera* extract and the components in WZ such as Schisandrol B and Schisandrin B against viral and chemical hepatitis and hepatic dysfunction induced by various chemical hepatotoxins [15–17, 19, 23, 24]. In addition, WZ exhibited an ability to increase GSH levels compared to the control. The elevated hepatic GSH levels by WZ are in agreement with previous studies showing that the hepatoprotective action of whole *Schisandra Sphenanthera* extracts is mediated by enhancement of GSH levels [17, 18, 23, 24]. However, at 2 h post-APAP treatment, both APAP- and WZ/APAP-treated mice exhibited extensive GSH depletion in the liver, though GSH in WZ/APAP-treated mice was partially restored. This shows that WZ cannot totally restore GSH status 2 h post-APAP treatment. Consistent with a previous study [8], hepatic GSH levels recovered at 24 h both in APAP- and WZ/APAP-treated mice. In addition to GSH, other biological studies have indicated that some schisandrins in WZ can induce heat shock proteins in mice to exert its hepatoprotective action [17]. However, the mechanisms underlying the hepatoprotective action of WZ against APAP-induce liver injury remain unknown. 

 In clinical practice, *Schisandra sphenanthera Fructus* whole extract was used for hepatoprotection. Chemical analysis of the whole extract revealed the presence of lignans with a dibenzocyclooctadiene skeleton such as Schisandrin A, Schisandrin B, Schisandrin C, Schisandrol A, Schisandrol B, Schisantherin A, and Schisantherin B [14]. The whole extract and some of the components in the extract were found to possess hepatoprotective effects against the hepatic dysfunction induced by various chemical hepatotoxins [15–18]. Structure-activity relationship (SAR) analyses suggested that the presence of a methylenedioxy group in the dibenzocyclooctadiene skeleton is an important structural feature for the hepatoprotective activities, and thus lignans with a methylenedioxy group such as Schisandrin B exhibit strong liver protective effect particularly under conditions of CCl_4_ intoxication [25]. However, in the current study, determination of the active components in WZ that plays the most important role in protection against APAP-induced liver injury was not established. To determine the potential active component of WZ in liver protection again APAP toxicity, it is crucial to measure the content of lignans in WZ. In our recent study, the content of major constituents in the extract including Schisantherin A, Schisandrin A, Schisandrin B, Schisandrin C, Schisandrol A, and Schisandrol B were determined using a validated LC-MS/MS analysis [26]. The results revealed that all the above-mentioned compounds were all detectable and were major constituents in WZ, but Schisantherin A and Schisandrin A were the most abundant components in WZ and other lignans such as Schisandrin B, C and Schisandrol A, B were much less than Schisantherin A and Schisandrin A. Some reports have proven the liver protective effect of Schisandrin B and Schisandrol B (Gomisin A) [16–18]. However, their levels in WZ were very low, and thus Schisandrin B and Schisandrol B may not have major roles in the protection of WZ against APAP hepatotoxicity. Schisandrin A was the second most abundant constituent in WZ, but it was reported that Schisandrin A did not protect against CCl_4_ hepatotoxicity due to lack of the methylenedioxy group [25]. Schisantherin A was the most abundant component in WZ. However, no studies on the effect of Schisantherin A on APAP hepatotoxicity have been reported. Recently, Schisantherin A was found to exhibit anti-inflammatory and antiproliferative activity [27, 28], whether its anti-inflammatory effect contributes to the hepatoprotection requires additional studies. For the complexity of the herbal extract, liver protective activity of WZ against APAP toxicity might be caused by a combination effect of all the active components in WZ and their potential active metabolites after administration. Therefore, further studies are needed to determine which active component in WZ plays the most important role in protection against APAP-induced liver injury.

 The molecular mechanisms involved in APAP toxicity are becoming clear. The toxicity event is initiated by formation of a reactive metabolite, which depletes GSH and binds to cellular proteins, leading to mitochondrial damage and subsequent damage to cell membranes and nuclei, as well as the disruption of cell death- and survival-related signaling pathways, leads to massive necrosis and apoptosis [1, 29]. Recently, a correlation between PPAR*α* and APAP hepatotoxicity was established as revealed by the protective effect of PPAR*α* activator against APAP-induced hepatotoxicity [30–32], and the importance of UCP2 in mediating this protective effects was firmly established [9]. Furthermore, inhibition of fatty acid *β*-oxidation was revealed as a major mechanism of the hepatotoxicity induced by APAP as evidenced by severe disruption of lipid metabolism and increase of acylcarnitines, triglycerides, and free fatty acids [8]. Here, WZ exhibited a significant protective effect against APAP-induced hepatotoxicity but the mechanism remains unclear. The question arises whether WZ has an influence in the fatty acid *β*-oxidation and how this effect plays a role in hepatoprotective effect of WZ against APAP toxicity. Therefore, a targeted metabolomic analysis was conducted on acylcarnitines to uncover the relationship among fatty acid *β*-oxidation, PPAR*α* activation, and hepatoprotective effect of WZ against APAP-induced toxicity.

 Long-chain acylcarnitines are the principal precursors of *β*-oxidation substrates and acylcarnitines in serum mainly come from the liver [33]. Results from previous studies clearly showed that serum palmitoylcarnitine levels, especially their kinetic profile, can provide predictive information for monitoring the initiation and progression of APAP-elicited hepatotoxicity [8, 9]. These observations were confirmed here, at 2 and 24 h after 400 mg/kg APAP treatment, oleoylcarnitine and palmitoylcarnitine levels were significantly higher than the corresponding control and the WZ/APAP-treated mice. These results are consistent with previous studies in which acylcarnitines were elevated early after APAP treatment and that their increase was indicative of early liver damage by APAP. To correlate acylcarnitine levels with the fatty acid metabolism in the WZ protection study, the levels of serum triglyceride (TG) and free fatty acid (FFA) were examined. Serum TG and FFA levels were consistent with the change in serum acylcarnitine levels 24 hours post-APAP dosing. Pretreatment with WZ prior to APAP administration blocked the increase in palmitoylcarnitine and oleoylcarnitine, and thus restored fatty acid *β*-oxidation to normal level. This observation further confirmed that APAP at 400 mg/kg can severely disrupt the *β*-oxidation of fatty acids while WZ can recover this inhibition and maintain the normal lipid metabolism. 

 Previous studies have revealed that inhibition of fatty acid *β*-oxidation through the suppression of PPAR*α* activation as a contributing mechanism of APAP hepatotoxicity, and PPAR*α* activation in *Cyp2e1*-null mice was much more significant and more persistent than its activation in wild-type mice following a toxic APAP dose, leading to more prolonged upregulation of PPAR*α* target genes (*Cpt1*, *Cpt2*, *Acot1*, and *Cyp4a10*) that are involved in the fatty acid *β*-oxidation pathway [8]. Thus, it was important to determine which among these target genes contributed to the hepatoprotective effect of WZ. In the present study, *Acot1* was strongly induced by WZ; similarly, the increased expression level of *Cpt1* was induced in WZ/APAP-treated mice and sustained through 24 h in WZ/APAP-treated groups while its expression in APAP-treated mice was reduced to basal level at the end of 24 h. These results indicate that significant and prolonged upregulation of *Cpt1* and *Acot1 *by WZ mainly contributed to maintaining normal fatty acid metabolism and thus potentially contributed to hepatic protection of WZ against APAP-induced hepatic toxicity.

 A recent study demonstrated that the PPAR*α* target gene UCP2 protects against elevated reactive oxygen species generated during APAP-induced hepatotoxicity [9]. Protection against hepatotoxicity by UCP2-induction through activation of PPAR*α* is associated with decreased APAP-induced c-jun and c-fos expression, decreased phosphorylation of JNK and c-jun, lower mitochondrial H_2_O_2_ levels, increased mitochondrial GSH in liver, and decreased levels of circulating fatty acyl-carnitines [9]. Whether UCP2, c-jun, c-fos, or JNK is associated with WZ-induced hepatoprotection against APAP toxicity needs to be addressed by further studies.

## 5. Conclusions

In summary, this study demonstrated significant hepatoprotection of WZ against APAP-induced liver injury and revealed that pretreatment with WZ prior to APAP administration blocks the increase in serum palmitoylcarnitine and oleoylcarnitine and thus restores fatty acid *β*-oxidation to normal level. The study further revealed that significant and prolonged upregulation of PPAR*α* target genes *Cpt1* and *Acot1 *by WZ mainly contributed to maintaining normal fatty acid metabolism and thus potentially contributed to the hepatic protection of WZ against APAP-induced hepatic toxicity. New insight into understanding the hepatoprotective effect of WZ against APAP-induced liver toxicity has thus been shown.
